# Muscle Contributions to L_4-5_ Joint Rotational Stiffness following Sudden Trunk Flexion and Extension Perturbations

**DOI:** 10.1155/2013/915428

**Published:** 2013-01-14

**Authors:** Joel A. Cort, James P. Dickey, Jim R. Potvin

**Affiliations:** ^1^Department of Kinesiology, University of Windsor, 401 Sunset Avenue, Windsor, ON, Canada N9B 3P4; ^2^School of Kinesiology, The University of Western Ontario, 1151 Richmond Street, London, ON, Canada N6A 3K7; ^3^Department of Kinesiology, McMaster University, 1280 Main Street West, Hamilton, ON, Canada L8S 4L8

## Abstract

The purpose of this study was to investigate the contribution of individual muscles (MJRS_m_) to total joint rotational stiffness (MJRS_T_) about the lumbar spine's L_4-5_ joint prior to, and following, sudden dynamic flexion or extension perturbations to the trunk. We collected kinematic and surface electromyography (sEMG) data while subjects maintained a kneeling posture on a parallel robotic platform, with their pelvis constrained by a harness. The parallel robotic platform caused sudden inertial trunk flexion or extension perturbations, with and without the subjects being aware of the timing and direction. Prevoluntary muscle forces incorporating both short and medium latency neuromuscular responses contributed significantly to joint rotational stiffness, following both sudden trunk flexion and extension motions. MJRS_T_ did not change with perturbation direction awareness. The lumbar erector spinae were always the greatest contributor to MJRS_T_. This indicates that the neuromuscular feedback system significantly contributed to MJRS_T_, and this behaviour likely enhances joint stability following sudden trunk flexion and extension perturbations.

## 1. Introduction

There is a complex arrangement of bones, ligaments, muscle, and nervous tissue which combine to maintain the structural integrity of the spine, thus reducing the potential for system buckling. For stability maintenance, Bergmark [1] identified the importance of the force distribution of the lumbar musculature. Other research has shown that passive tissues of the lumbar spine can only provide minimal resistance to compressive loads (up to 90 N), thus the majority of stiffness is provided by the muscles, demonstrating the importance of muscles for joint safety [2]. Moorhouse and Granata [3] and Sinkjaer et al. [4] stated that involuntary muscle force contributions account for 35 to 42% of the total joint stiffness following a perturbation. Although muscles are vital for joint safety, their force distribution relies on the careful control of the nervous system to properly coordinate the required joint stiffness. Poor neuromuscular coordination has been suggested to be a risk factor for mechanical failure following kinematic disturbances [5–9]. Granata and England [10] were among the first to characterize the neuromuscular control of stability during *dynamic* trunk flexion/extension movements. However, that research did not account for scenarios where the *timing* of trunk disturbances was unknown and, thus, the results cannot be used to explain the implications of the common scenario of an unexpected kinematic disturbance, such as a slip or shift in load, where involuntary muscleforce contributions are crucial.

Numerous studies have contributed to our understanding of lumbar spine stability; however, there are limits to the conclusions about stability due to the majority of these studies either quantifying joint stability during static conditions [11–15], using theoretical and mathematical concepts [16–19], utilizing in vitro techniques [20–25] or approximated joint stability using electrophysiology combined with joint kinematics [26–30]. Furthermore, of the studies that calculated stability, only net joint stability throughout the motion was reported without information detailing the individual muscle contribution to stability [11–15]. In must be noted that Brown and Potvin [17] calculated individual muscle contributions to joint rotational stiffness (MJRS); however, since empirical-based data were not used in this work, only theorically based results were provided. Thus, there is a need for further research of the role that the neuromuscular system plays in maintaining stability in response to a sudden perturbation, through the control of individual muscles. However, in order to understand these roles, it is imperative that the complexities caused by the interaction between the skeletal and neuromuscular systems are minimized. Specific to the lumbar spine, to limit such interactions the sudden perturbations should cause joint motion about the flexion/extension axis given that rotation about this axis presents less of a challenge to the neuromuscular system based on the symmetrical design of the bilateral flexor and extensor musculature. This type of study design will provide for an initial and basic understanding of how the neuromuscular system aids in joint stability of the lumbar spine. Detail at this level can contribute to furthering our understanding of how various modes of joint instability can ultimately contribute to injury risk [17].

The purpose of this research was to investigate the contribution of the trunk muscles to joint rotational stiffness about the lumbar spine's L_4-5_ joint prior to, and following, sudden dynamic flexion and extension perturbations to the trunk. In particular, this project examined the sum of all muscles contributing to the total MJRS (MJRS_T_), as well as the contribution of individual muscles to MJRS_T_ (MJRS_m_). It was hypothesized that prior knowledge of both perturbation timing and direction would be accompanied by increased MJRS_T_ prior to the perturbation, resulting in decreased trunk motion. In addition, it was hypothesized that prior knowledge of the perturbation direction would cause a neuromuscular strategy such that individual muscle contributions to MJRS_T_ would be dependent upon the forced direction.

## 2. Methods

### 2.1. Subjects

This study included 7 male subjects with a mean age of 24.7 ± 2.4 years, height of 178.5 ± 4.6 cm, and mass of 77.0 ± 8.5 kg. All subjects were free of musculoskeletal injury to the trunk, neck, and upper limbs. The University's Research Ethics Board approved all aspects of the study.

### 2.2. Instrumentation and Data Acquisition

We collected fourteen channels of surface electromyography (sEMG), using the placement protocol outlined in Cholewicki and McGill [31], bilaterally for the following muscles: rectus abdominis (RA), external oblique (EO), internal oblique (IO), lumbar erector spinae (LES), thoracic erector spinae (TES), multifidus (MULT), and latissimus dorsi (LD). We positioned disposable bipolar Ag-AgCl surface electrodes (Medi-trace disposable electrodes, Kendall, Mansfield, MA) in an-orientation parallel to each muscle's line of action, between the myotendinous junctions and innervation zones as per Shiraishi et al. [32]. The interelectrode distance was 2.5 cm. We collected and amplified the sEMG signals using two Bortec AMT-8 systems (Bortec Biomedical, Calgary, Canada, 10–1000 Hz, CMMR = 115 dB, gain = 500–1000, input impedance = 10 GΩ). We A/D converted these signals at a sample rate of 2000 Hz using a 16-bit A/D converter (ODAU II, Northern Digital Inc., Waterloo, Canada).

We collected kinematic data using an active marker system (Optotrak 3020, Northern Digital Inc., Waterloo, Canada) sampling at 100 Hz. We placed two marker arrays on rigid fins, each with four infrared emitting diodes, and rigidly secured them to the midline of the body at the pelvis (middle of sacrum), representing the lumbar region, and rib cage (approximately at T9 level), representing the thoracic region. We used a parallel robotic platform (R2000 Rotopod, PRSCo, NH, USA) to apply the sudden inertial trunk flexion or extension perturbations. Finally, to measure acceleration and timing of the platform perturbations, we attached a triaxial accelerometer (Crossbow CXL75M3, Crossbow Technology Inc., Milpitas, CA) to the robotic platform and sampled the data at 2000 Hz.

### 2.3. Experimental Procedures and Protocol

Prior to the experimental trials, subjects performed isometric maximal voluntary exertions (MVEs) for each muscle to be later used to normalize the sEMG data collected during experimental trials. To obtain the MVE of the abdominals (RA, IO, and EO), subjects laid in a supine position, replicating a “sit-up” position with the feet braced to ground, and performed a sequence of isometric maximal trunk flexion efforts that also included twist and lateral bend efforts, against the resistance of the researchers. The subjects performed the MVEs for the trunk extensor muscles (LES, TES, LATS, MULT) while lying in a prone position with the feet braced, and subjects executed a sequence of maximal trunk extension efforts, against resistance manually applied by the researchers. Each of the abdominal and back muscle efforts were isometrically held for 2-3 seconds and 30 second rests were provided in between each of the efforts.

**Figure 1:**
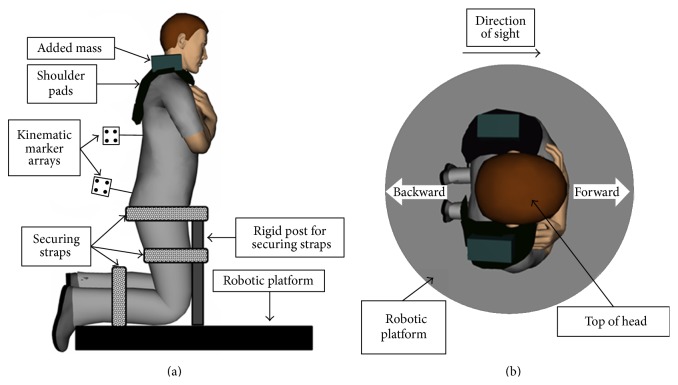
An illustration of the experimental device in a sagittal (a) and a coronal view (b). Subjects knelt on the robotic platform and legs (below the pelvis) were secured to framing that was attached to the platform. Subjects wore modified shoulder pads and maintained an upright neutral trunk posture with both arms crossed in front of the chest.

After this, we positioned the subjects in a kneeling posture on a robotic platform and harnessed them into an apparatus that minimized motion below the pelvis, but allowed for unconstrained motion of the trunk and head. Also, subjects crossed their arms in front of their chest to minimize motion of the upper limbs and to maintain an erect trunk posture (Figure 1). The parallel robotic platform applied the sudden inertial trunk flexion or extension perturbations, through rapid linear anterior or posterior 4 cm displacements of the platform (peak accelerations = 4 m/s/s). Preexperimental testing showed that the perturbation profiles were sufficient to elicit an electromyographic response. 

We exposed each subject to 16 perturbation conditions, which included two timing-knowledge conditions and two direction-knowledge conditions in four perturbation directions, assigned in a random order. The timing knowledge conditions were (1) known timing (KT) and (2) unknown timing (UT). The perturbation device was equipped with dual controls such that it could be engaged manually by the subject during the KT conditions, via an electronic trigger button, or through computer activation using a digital trigger signal for UT conditions. During UT conditions, we informed the subjects of the start of the trial; however the computer randomly assigned a time to engage the perturbation device within a 15-second period after the informed start. The directional knowledge conditions were (1) known direction (KD) and (2) unknown direction (UD). The different perturbation directions were forced trunk: (1) flexion via posterior linear platform displacements (*P*_FLEX_), (2) extension via anterior platform displacements (*P*_EXT_), (3) left lateral bend via right platform displacements, and (4) right lateral bend via left platform displacements. Only data from the forced flexion and extension trials will be discussed in this paper. To enhance the effect of the perturbations, we rigidly attached modified football shoulder pads to the trunk that allowed us to add mass to the trunk via evenly distributed fixed weights to each shoulder (15% of each subject's upper body mass, including head, trunk, and upper extremities taken from [33]).

### 2.4. Data Analysis

We conditioned all sEMG data by removing the DC bias, high pass filtering at 140 Hz (Butterworth, 6th order) [34, 35], rectifying, low-pass filtering at 2.5 Hz (Butterworth, 2nd order) and normalizing to the MVE. In addition, we used the thoracic and lumbar kinematic marker arrays to determine the relative angle of the trunk. Specifically, the thoracic segment was defined by the marker array that was fixed to the spinous process at T9 and the lumbar segment was defined by the marker array attached to the sacrum (described in Section 2.2). Using this method the trunk angle was calculated as the intersection of the line connecting the thoracic and lumbar marker arrays [36]. The lumbar angle was represented as a fraction of the total trunk angle. For each of the orthogonal axes, the following percentages represent the lumbar component of the overall angle: flexion = 72.2%, extension = 43.5%, lateral bend = 49.1%, and axial twist = 5.6% [37–39]. Furthermore, the L_4-5_ joint angle was represented as a fraction of the total lumbar angle. The L_4-5_ component of the overall lumbar angle for each axis are as follows: flexion = 22.4%, extension = 9.5%, lateral bend = 16.2%, and axial twist = 13.3% [37–39]. We processed the joint angles with a critically damped dual-pass Butterworth filter with a final cut-off of 5 Hz (2nd order). The trunk angles were reported as the calculated displacement from the resting sitting angle to the peak angle following the perturbation. Also, we dual lowpass Butterworth filtered the tri-axial accelerometer data using a 50 Hz cutoff. Following conditioning, we downsampled all signals to 100 Hz.

We utilized the normalized and conditioned instantaneous bilateral sEMG and joint angle data as inputs to a biomechanical trunk model developed by Cholewicki and McGill [31], to determine muscle forces and moments. These data were used to calculate MJRS_T_ about L_4-5_ about the flexion/extension, lateral bend, and axial twist axes. Specifically, the Cholewicki and McGill [31] kinematic lumbar spine model was utilized in this study to determine the kinematics of each muscle's instantaneous length, velocity, and moment arm. We used the normalized and conditioned instantaneous sEMG data as input into this model to provide a first approximation of instantaneous muscle force based on each muscle's sEMG (normalized to MVE), instantaneous muscle length (as per [40]), velocity (as per [41]), and maximal muscle stress set at 1 N/cm^2^. While common estimates of muscle stress typically fall within the range of 30–100 N/cm^2^, the actual magnitude of this variable was not a critical component of the current calculation since the focus of this study was to examine the contribution of individual muscles as percentage of a theoretical maximum MJRS_T_, which is described in more detail in a later paragraph. Thus, the maximum muscle stress value was arbitrary as it was held constant (value of 1) during the sEMG-muscle force modelling between the theoretical maximum and the experimental conditions.

**Figure 2:**
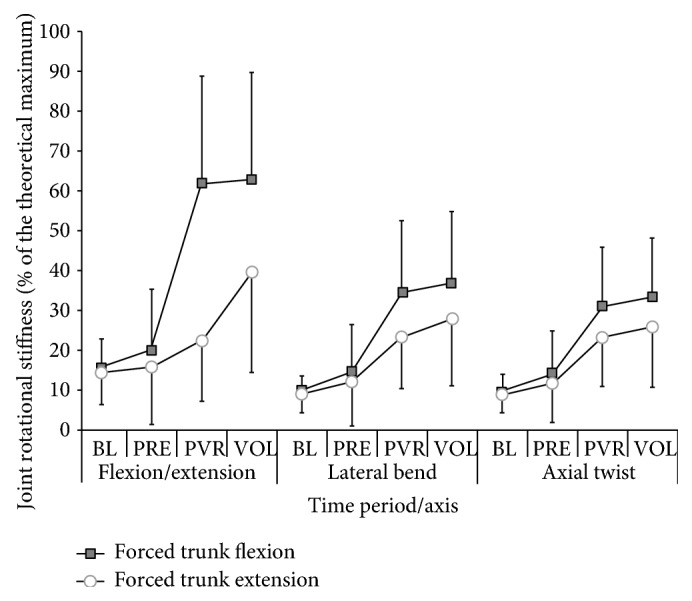
The MJRS_T_ (as a percentage of the theoretical MJRS maximum) is shown by time period for each axis of the three axes. Displayed is the MJRS_T_ for each axis for both the forced trunk flexion and forced trunk extension. Included in the graph are the standard deviations for each of the data points.

We utilized the equation of Potvin and Brown [19] to calculate the MJRS_m_ about the three orthopaedic axes of the L_4-5_ joint. In this study, a constant relating muscle force to muscle stiffness (*q*) was set to 10 as recommended by Potvin and Brown [19]. The *q* value was further corrected to account for muscle contraction velocity, as Cholewicki and McGill [42] found that muscle stiffness decreases as muscle contraction velocity increases (both concentrically and eccentrically). We developed regression equations (*r* ^2^ = 0.99) based on the stiffness curve in Figure 2 of Cholewicki and McGill [42], such that outputs from these equations modulated each muscles *q* value to accommodate the effects of contraction velocity. The muscle stiffness corrections were then multiplied by the constant *q* value for each muscle's instantaneous contraction velocity. For each muscle, the MJRS equation then used the estimated muscle forces, described above, and the geometric orientation of the muscles and their nodes, to calculate MJRS_m_ values about each of the three axes.

The summation of all individual MJRS_m_ contributions within each respective axis, at each instant in time, allowed us to determine the MJRS_T_. Rather than reports the actual estimated MJRS_m_ and MJRS_T_ values, we normalized these values as a percentage of the theoretical maximum MJRS_T_ when the trunk was presumed to have maximal stiffness in the upright neutral posture (0 degree trunk flexion angle). Specifically, we calculated muscle kinetics using the previously described modelling methodology; however, we used the theoretical sEMG values in place of experimentally recorded data. We assigned an activation of 100% MVE to the RA, IO, and EO muscles, of the weaker trunk flexor muscle group, and then we calculated the activation of the stronger trunk extensor group (LES, TES, MULT, and LATS), necessary to balance the moment about the flexion/extension axis to zero. We used these theoretical activations to calculate the individual muscle forces, assuming a maximal muscle stress of 1 N/cm^2^, and subsequent MJRS_m_ and MJRS_T_ values about each of the three axes. We considered these MJRS_T_ values as the maximum theoretical magnitudes about each axis and used them normalize all previously estimated experimental MJRS_m_ values as a percentage of maximum theoretical value within each axis.

We windowed the MJRS_T_ MJRS_T_ and MJRS_m_ data into four time periods based on Stokes et al. [29]: (1) baseline (BL) from 500 to 450 ms prior to the perturbation, (2) preperturbation (PRE) from the 50 ms prior to the perturbation, (3) prevoluntary response period (PVR) from 25–150 ms after perturbation (incorporating both short and medium latency neuromuscular responses), and (4) voluntary response period (VOL) from 150 to 300 ms after perturbation. We calculated the mean and standard deviations for MJRS_T_ and MJRS_m_ during BL and PRE. To ensure that the full response of the system was captured following the perturbation, we determined the individual peak MJRS_T_ values within each of the PVR and VOL time periods.

Finally, the sEMG onset was used to estimate the timing of each muscle amplitude change following the perturbations [29, 43]. For each trial and muscle, sEMG onset was determined using the integration method of Santello and McDonagh [44] and manually confirmed based on the threshold method described by Hodges and Bui [43]. We removed any onset timing data from the analysis if the detected onset occurred 400 ms after the perturbation, based on work by Wilder et al. [45], who found that muscular responses that occurred 400 ms or more after a perturbation are not a direct result of the perturbation.

### 2.5. Statistical Analysis

For all 8 conditions, within each subject, we calculated means and standard deviations for each dependent variable across the five repeated trials. We used these mean values to represent each subject's response to that condition within the subsequent statistical analysis. A 2 × 2 × 2 × 2 × 4 analysis of variance (ANOVA), with repeated measures, was used to determine the influence of each of the five independent variables: muscle side location (left and right), time knowledge (KT and UT), perturbation direction (*P*_EXT_ and *P*_FLEX_), and direction knowledge (KD and UD), as well as time period (BL, PRE, PVR, and VOL). The significance level for each ANOVA was set at *P* < 0.05. The dependent variables for this analysis included MJRS_T_ and MJRS_m_ for each muscle. For the significant main and interaction effects, we compared means with a Tukey's HSD post hoc test. We also used an *ω* ^2^ analysis on each statistical interaction to calculate the percentage of the total variance explained by the interaction. To be considered for discussion, we required all interactions to account for at least 1% of the total variance [46, 47]. In addition, a 2 × 2 ANOVA, with repeated measures, was used to determine the effect of perturbation direction and direction knowledge on the sEMG onset timing (excluding KT data) dependent measure. We used the same post hoc test and *ω* ^2^ analysis as described above on the statistical analysis for this dependent measure.

## 3. Results

The results of the dependent measures from this study are detailed within this section. To better understand the magnitude of the perturbations, we have included the calculated joint angles and accelerations for the trunk and L_4-5_ for each axis in Table 1.

### 3.1. Total L_4-5_ Joint Rotational Stiffness

The total theoretical maximum MJRS_T_ was 412, 419, and 241 Nm/rad for the FE, lateral bend, and axial twist axes, respectively (Figure 2). For all 3 axes, there was a significant interaction between time period and perturbation direction (F/E *P* < 0.001, lateral bend *P* < 0.01, and axial twist *P* < 0.01). Post-hoc analysis showed that, for the F/E axis during the forced flexion, the MJRS_T_ increased as the time period progressed from BL to PVR, BL to VOL, PRE to PVR, and PRE to VOL. Also the post-hoc analysis revealed that, during the forced extension, MJRS_T_ increased from BL to VOL, PRE to VOL, and PVR to VOL. For both the lateral bend and axial twist axes, in both the forced flexion and extension conditions, MJRS_T_ increased from BL to PVR, BL to VOL, PRE to PVR and PRE to VOL. Interestingly, the direction knowledge variable did not significantly influence MJRS_T_ for any of the 3 axes.

### 3.2. Individual Relative Muscle Contributions of Total Joint Rotational Stiffness

**Figure 3:**
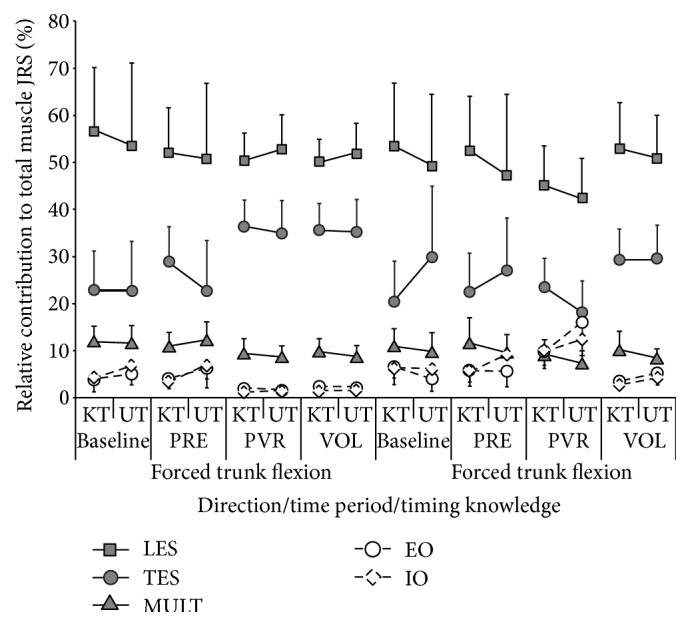
The MJRS_m_ data is shown for each orthogonal axis when subjects both possessed and did not possess perturbation timing awareness. In addition, these data are also separated into each of the experiment time period classification. Included in the graph are the standard deviations for each of the data points.

We calculated the muscle contributions to MJRS_T_ about each orthogonal axis; however, only contributions about the F/E axis will be presented, as it is the primary axis about which the perturbation acted (Figure 3). There was no significant effect of muscle side, indicating symmetrical trunk motion, so we averaged data from the left and right sides for each muscle. Also, we assumed that changes of less than 2% of MJRS_T_ were not functionally relevant and, thus, only significant (*P* < 0.05) effects, with average differences greater than 2% of MJRS_T_, are presented. The RA and LATS were the only muscles that did not ever meet this requirement.

There was a significant three-way interaction between time period, perturbation direction, and timing knowledge for the EO muscle (*P* < 0.001). Further post-hoc analyses revealed no differences between the known and unknown timing within any of the time periods during the forced flexion. However, during the forced extension trial, KT was higher than UT at PRE and UT was higher than KT at PVR. There also was a significant interaction between time period and perturbation direction (*P* < 0.0001). During the *P*_FLEX_ condition, we found a significant decrease in IO's relative contribution to MJRS_T_ from both BL and PRE to both PVR and VOL. During the *P*_EXT_ conditions, there was an increase as time periods advanced from BL and PRE to PVR and significantly lower values at VOL than at both PRE and PVR. Finally, direction knowledge did not significantly influence the response of any of the trunk flexor muscles.

The relative contribution of LES to MJRS_T_ had a significant interaction between time period and perturbation direction (*P* < 0.05). There were no differences between time periods for the *P*_FLEX_ condition. However, for the *P*_EXT_ condition, the PVR values were lower than those at BL, PRE and VOL. The TES contribution to MJRS_T_ had a 3-way interaction between time period, perturbation direction, and timing knowledge (*P* < 0.05). Although there were no differences found in the *P*_FLEX_ data, UT was higher than KT at BL for the *P*_EXT_ condition. Also, there was a main effect of time period for the MULT MJRS_T_ contribution (*P* < 0.05). Post-hoc analyses showed a 27% decrease in contribution as time period advanced from BL to PVR and PRE to PVR. Lastly, direction knowledge did not significantly influence the response of any of the trunk extensor muscles.

### 3.3. sEMG Onset Timings

**Figure 4:**
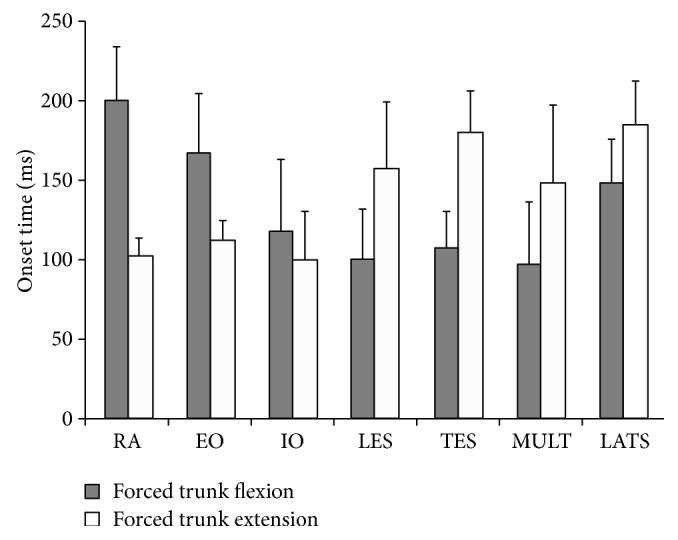
The mean and standard deviations of the sEMG onset timings for each recorded muscle (ms).

Main effects of perturbation direction for sEMG onset timing were found for all muscles, except for IO (Figure 4). Specifically, the onset times for EO and RA were higher in the *P*_EXT_ compared to the *P*_FLEX_ condition (*P* < 0.01 and *P* < 0.001 resp.), and both the RA (*P* < 0.001) and EO (*P* < 0.01) had later onset times. The LES, TES, MULT, and LATS showed a main effect of perturbation direction (*P* < 0.001, *P* < 0.0001, *P* < 0.01, *P* < 0.05, resp.), and post-hoc analyses showed that onset times were higher for these muscles in the *P*_EXT_ compared to the *P*_FLEX_ condition. In addition, for the MULT muscle, we found the UD onset times to be 10% higher than for KD (*P* < 0.05).

## 4. Discussion

The purpose of this research was to investigate trunk muscle contributions to joint rotational stiffness about the lumbar L_4-5_ joint prior to, and following, sudden inertial flexion and extension perturbations to the trunk. Our unique perturbation methodology allowed for us to determine that possessing the knowledge of perturbation direction does not affect MJRS_T_, whereas awareness of the perturbation timing does cause an increase in MJRS_T_ magnitude. In addition, based on our knowledge this is the first work that determined individual muscle contributions to joint rotational stiffness, prior to and following sudden trunk perturbations. Based on our work we found that the LES was the greatest contributor to MJRS_T_, followed in order by the TES, MULT, EO, and IO.

We also found that the response of the neuromuscular system, immediately following forced trunk flexion and extension, was a significant contributor to MJRS_T_, which supports previous research findings. In our work the greatest MJRS_T_ magnitude was always about the flexion/extension axis, followed by the lateral bend and axial twist axes. Since the F/E axis was the primary contributor to MJRS_T_ in the current study, the remainder of this discussion will focus on that axis.

Our work suggests that it is most likely that the prevoluntary response, incorporating both short and medium latency neuromuscular responses, was an attempt to limit the perturbation motion. It served as a first responder, initially providing stiffness until the voluntary component began its contribution. Albeit smaller in magnitude, this prevoluntary response likely plays a critical role in injury avoidance, given that the voluntary response may not occur early enough after the perturbation.

### 4.1. MJRS_T_: Timing Knowledge

The MJRS_T_ increased when the subjects knew the perturbation timing, demonstrating that timing awareness promoted increased joint rotational stiffness. This finding is consistent with previous studies that identified that subjects increased muscle activation and, thus joint stiffness, prior to the perturbation [27–30, 48, 49].

A deeper investigation of our data showed that, with timing knowledge, most subjects tended to increase MJRS_T_ from the baseline measure to just prior to the perturbation (PRE). This suggests an anticipatory adjustment in preparation for the forced motion. However, there were two subjects who, during each of the known timing-trunk extension trials, showed increased MJRS_T_ magnitudes during the PRE and PVR time periods with respect to the values calculated during the baseline periods. While this approach may provide maximum safety against the expected perturbation, it is also metabolically inefficient to maintain elevated muscle activity for unnecessarily long-time periods.

### 4.2. MJRS_T_: Direction Knowledge

The robotic device allowed for multidirectional forced motion. This device enabled a unique inertial perturbation approach, compared to most previous experimental protocols used for sudden loading studies where a harness-cable system has been used to perturb subjects. Given that the required cable used in such a system to pull the body segment to produce the perturbation provided subjects with knowledge of the perturbation direction, only timing knowledge could be manipulated. Our robotic platform also allowed for increased uncertainty with regard to the direction of the perturbation. Nevertheless, the results revealed that direction knowledge did not affect the neuromuscular response to trunk perturbations. This was unexpected as we had hypothesized that the awareness of direction, like that seen for timing knowledge, would offer assistance to the neuromuscular system for coordinating the recruitment of muscle forces for increased MJRS_T_.

To the best of our knowledge, this is the first published sudden trunk loading research that incorporated conditions where the perturbation direction was completely unknown to the subject. Masani et al. [50] completed a multidirectional perturbation study of the trunk and found that muscle responses were dependent upon the forced direction; however, their subjects were always aware of the perturbation direction. Cholewicki and VanVliet [14] showed that loading direction affects the contribution of individual muscles to joint stability during isometric trunk exertions; however, the preexisting data does not provide details on whether such coordination occurs in preparation for an unexpected disturbances. It is possible that it may be difficult to prioritize specific individual muscle recruitment for optimal joint rotational stiffness, in preparation for sudden motion. Brown et al. [51] found that cocontraction (abdominal muscle force during forced trunk extension) increased trunk stiffness prior to a sudden perturbation; however, their subjects lacked the ability to selectively increase abdominal muscle force without a subsequent increase in back muscle activity, which potentially increases the risk of injury given the subsequent increase in trunk compressive forces.

### 4.3. MJRS_m_ General Considerations

Of the seven bilateral muscles recorded and modeled, the RA and LATS did not meet the statistical requirements, discussed previously, to be considered significant contributors to MJRS_T_ in the context of this research. However, the IO, EO, MULT, LES, and TES all contributed to MJRS_T_, albeit at various levels.

A qualitative comparison of each muscle's contribution showed that the LES was the greatest contributor followed, in order, by the TES, MULT, EO, and IO (see Figure 3). This order of muscle contribution is reflected in other similar studies, such as Chiang and Potvin [27], Krajcarski et al. [28], and Thomas et al. [30]. These findings demonstrate that no one muscle is exclusively responsible for generating joint rotational stiffness, but that it is a collection of muscles acting together to generate the required resistance. Furthermore, both Brown and Potvin [17] and Crisco and Panjabi [52] suggest that the “global” multisegmental muscles, which possess larger moment arms, are the main contributor to joint rotational stiffness. This concept is supported by the current work where the primary contributors to MJRS_T_, LES, and TES have the longest moment arms.

During the forced extension conditions, we expected that the IO and EO muscles would be the main contributors to MJRS_T_, since they acted as antagonists during the motion. However, this was not the case and may be a result of the relatively small trunk extension motion that was caused by the perturbation. This is a limitation in our study. The magnitude of the extension perturbation was set to a level that would have minimal risk of injury; however, this may have been insufficient to elicit substantial length changes for the abdominal muscles and cause them to activate.

### 4.4. MJRS_m_: Timing Knowledge, Direction, and Direction Knowledge Interaction

The TES and EO were unique in that their contributions were dependent on all of the experimental variables (timing knowledge, direction of the forced motion, and time period). During the unexpected timing conditions, when forced into trunk extension, there was a greater relative contribution from the EO just prior to the perturbation. In the same experimental condition, the EO greatly increased its relative contribution to MJRS_T_ during the prevoluntary time period, when timing knowledge was not provided. Vera-Garcia et al. [53] found similar EO response patterns during unanticipated trunk extension perturbations; however, when subjects anticipated the perturbation, as seen through increased voluntary contraction of the other monitored muscles, the EO response was significantly reduced. For the TES, timing knowledge only impacted the baseline time period, with no muscle contribution changes observed just prior to, or following, the perturbation. As such, these results are considered to be functionally irrelevant and are likely due to slight adjustments in trunk posture at the start of the trials.

The behaviour of the EO is likely the result of increased magnitudes of MJRS_T_ associated with the anticipation of the perturbation. Specifically, in the presence of timing awareness, the anticipatory activity of this muscle raised the magnitude of its MJRS_T_. Accordingly the joint became stiffer prior to, and throughout, the forced motion. This ultimately allowed for less dependence on the prevoluntary contribution. Thus, in order to obtain the necessary levels of stiffness, a feed-forward neuromuscular strategy was utilized reducing the dependency on the involuntary muscle response as seen during the unexpected timing conditions.

Qualitative examination of the individual muscle contributions to MJRS_T_ revealed that the antagonist muscles (those muscles not involved in arresting the forced motion) were active both prior to (PRE), and following (PVR and VOL), the perturbation. Rather than aiding in arresting the forced motion, it is likely that these muscles are utilized to increase L_4-5_ joint's overall rotational stiffness, and thus joint safety, at the expense of greater moment in the direction caused by the perturbation. However, this increase in joint moment caused by the cocontracting muscles may be a necessary “tradeoff” to ensure adequate joint stiffness. Increased muscle forces of the trunk through cocontraction are thought to be important for stiffness of the spine, which ultimately aids in stabilizing the joint [54, 55].

As mentioned earlier, reliance on the feedback mechanism, when timing awareness is not available, may be intended to optimize the balance between tissue loading and joint stiffness. Granata and Marras [54] noted that there is a “tradeoff” between tissue loading and spine stability; a balance is needed in order for lumbar spine motions to occur with minimal risk of injury. A strategy of muscle preactivation, in anticipation of a kinematic disturbance, results in greater muscle forces (although not calculated in this study), and may cause higher compressive loads on the spine [27, 51, 53, 54]. These higher compressive loads are important since high compressive forces are a risk factor for low back injury [56].

It must be noted that only the EO and TES were affected by the relationship between timing awareness and time period, whereas the remaining muscles were not affected by this relationship. Similar to the findings for MJRS_T_, we have concluded that some subjects tended to increase their levels of muscle activation right from the beginning of the trial (starting at BL) through to the end. However, not all subjects employed this approach and due to this, we have hypothesized that those having timing awareness, that showed increased responses following the perturbation (and thus minimal pre-perturbation muscle anticipation), were exhibiting physiologically efficiency, as they would have been required to maintain higher levels of muscle activation for extended periods of time. Therefore, those subjects showed that it is more physiologically economical, in cases where timing was unknown, to begin activation just prior to the perturbation, while maintaining joint rotational stiffness.

## 5. Conclusions

Although the magnitudes of the prevoluntary muscle forces are smaller than those produced voluntarily, our data suggests that subjects adopted a response strategy that relies on prevoluntary (reflex) muscle forces to produce rapid increases in joint rotational stiffness following a perturbation. Findings from this study support those of Moorhouse and Granata [3], Granata and England [10] and Sinkjaer et al. [4], as these authors observed that prevoluntary muscle force contributions are important to joint integrity during either simple voluntary trunk motion or following sudden trunk perturbations. Our work shows that a strategy that includes MJRS from the reflex response could be considered superior since an immediate but lower magnitude response allows the system to safely increase joint stiffness, rather than deferring the full responsibility later in time to the voluntary response. Based on this work, it is apparent that the early muscle response plays a vital role in joint safety during sudden kinematic disturbances. These findings can be used to better understand the role of the neuromuscular system during sudden trunk perturbations, both when timing and direction knowledge are varied.

## Figures and Tables

**Table 1:** Summary of the mean and standard deviations of the joint angle and acceleration magnitudes prior to (BL time period) and following the perturbations (VOL time periods). The BL angles and accelerations were calculated as the average magnitudes during that time period, whereas the peak magnitudes found during the VOL time period are reported.

Measure	Trunk			L_4-5_	
	Axis	BL	VOL	BL	VOL

Joint angle (degs)	Flex/Ext	3.7 ± 2.3	5.6 ± 2.5	0.7 ± 0.8	1.1 ± 0.9
	Lat. bend	1.8 ± 1.1	2.3 ± 1.0	0.2 ± 0.1	0.2 ± 0.1
	Twist	1.3 ± 1.1	2.0 ± 1.1	0.1 ± 0.1	0.2 ± 0.1

Joint acceleration (degs/s/s)	Flex/Ext	7.1 ± 27.8	336.3 ± 122.7	1.2 ± 5.8	51.7 ± 31.8
	Lat. bend	3.8 ± 4.5	70.3 ± 25.0	0.4 ± 0.5	7.5 ± 2.7
	Twist	5.8 ± 6.2	66.8 ± 23.4	0.6 ± 0.7	7.4 ± 2.6
